# Supplementary material for: GBStools: A Statistical Method for Estimating Allelic Dropout in Reduced Representation Sequencing Data
Source: PLoS Genet. 2016 Feb 1;12(2):e1005631. doi: 10.1371/journal.pgen.1005631 (PMC4734769; doi:10.1371/journal.pgen.1005631)
Supplement: S1 Text — (PDF) [file pgen.1005631.s001.pdf]

S1 Appendix. Allele frequency estimation in GBS data sets by expectation-maximization

March 24, 2015

1 Notations

Suppose there are N sites for n diploid individuals, and each site is composed of a restriction site with alleles {+, -}, and a SNP with alleles {A, a}. SNP alleles on the same haplotype as the '+' allele are sampled by GBS, but alleles on the same haplotype as the '-' allele are not. The '-' allele (and any 'A' or 'a' allele associated with it) cannot be observed directly, but can be observed indirectly because reduced sampling causes reduced sequencing coverage. Therefore let {A, a, -} be the set of observable alleles, and let {AA, Aa, aa, A-, a-, - -} be the set of observable genotypes.

Let $\mathbf{G} = (\vec{G_1}, ..., \vec{G_N})^\intercal$ be the observable genotypes with vector $\vec{G_s} = (\vec{G_{s,1}}, ..., \vec{G_{s,n}})$ representing the observable genotypes at site s, and $G_{s,i,1}$, $G_{s,i,2}$, $G_{s,i,3}$ representing the number of 'A', 'a', and '-' alleles for individual i. For convenience, we may drop the position subscript s when we are looking at only one locus. Let $\vec{\phi} = (\phi_1, \phi_2, \phi_3)$ be the site allele frequencies for the observable alleles.

Let $\mathbf{D} = (\vec{D_1}, ..., \vec{D_N})^\intercal$ be the data matrix with vector $\vec{D_s} = (\vec{D_{s,1}}, ..., \vec{D_{s,n}})$ representing the read data at site s. Let $\vec{d_s} = (|\vec{D_{s,1}}|, ..., |\vec{D_{s,n}}|)$ be a vector of the number of reads for each sample. Let λ be the site mean coverage for samples with genotypes {(2, 0, 0), (1, 1, 0), (0, 2, 0)} (i.e. for '++' samples).

Let $\mathbf{Z} = (\vec{Z_1}, ..., \vec{Z_N})^\intercal$ be a matrix of variables indicating success (1) or failure (0) of the restriction digest, where $\vec{Z_s} = (Z_{s,1}, ..., Z_{s,n})$. If $Z_{s,i} = 0$, then $d_{s,i} = 0$ regardless of the genotype of the i-th sample. Let δ be the site failure rate.

2 Estimating the site allele frequency

We aim to find $\vec{\phi}$, λ, and δ that maximize $\Pr\{\vec{D}|\vec{\phi}, \lambda, \delta\}$. We have:

$$\log \Pr\{\vec{D}, \vec{g}, \vec{z}|\vec{\phi}, \lambda, \delta\} = \log \prod_{i=i}^{n} \Pr\{\vec{D}_i|g_i, d_i\} \Pr\{d_i|g_i, z_i, \lambda\} \Pr\{g_i|\vec{\phi}\} \Pr\{z_i|\delta\}$$

$$= \log \prod_{i=i}^{n} \prod_{j=1}^{d_i} \Pr\{D_{i,j}|g_i\} \Pr\{d_i|g_i, z_i, \lambda\} \Pr\{g_i|\vec{\phi}\} \Pr\{z_i|\delta\}$$

$$= C + \sum_{i=i}^{n} \log \Pr\{d_i|g_i, z_i, \lambda\} \Pr\{g_i|\vec{\phi}\} \Pr\{z_i|\delta\}$$

Let $m_i = 2 - g_{i,3}$ be the observable ploidy for the i-th individual (i.e. the number of '+' alleles it carries), and let $\vec{r} = (r_1, ..., r_n)$ be a vector of read count normalization factors, where

$$r_i = \frac{\sum_{s=1}^{N} d_{s,i}}{\frac{1}{n} \sum_{j=1}^{n} \sum_{s=1}^{N} d_{s,j}}$$

We assume that the sample read count, d_i follows a negative binomial distribution with mean $\mu = \lambda z_i r_i \frac{m_i}{2}$ and size parameter ψ:

$$\Pr\{d_i|g_i, z_i, \lambda\} = \frac{\Gamma(d_i + \psi)}{\Gamma(d_i + 1)\Gamma(\psi)} \left(\frac{\psi}{\lambda z_i r_i \frac{m_i}{2} + \psi} \right)^{\psi} \left(\frac{\lambda z_i r_i \frac{m_i}{2}}{\lambda z_i r_i \frac{m_i}{2} + \psi} \right)^{d_i}$$

Let $disp(\mu) = a\mu + 1$ be a function chosen to model the dispersion in the normalized read counts, $\vec{d} \circ \vec{r}$. The negative binomial variance is $\mu + \frac{\mu^2}{\psi}$. Therefore ψ is constant across all N sites and $\psi = \frac{1}{a}$.
We assume Hardy-Weinberg equilibrium for the observable genotypes:

$$\Pr\{G_i = g_i|\phi\} = \binom{2}{g_{i,1}, g_{i,2}, g_{i,3}} \phi_1^{g_{i,1}} \phi_2^{g_{i,2}} \phi_3^{g_{i,3}}$$

And the probability of the digest success/failure state for the i-th individual is:

$$\Pr\{Z_i = z_i|\delta\} = (1 - \delta)^{z_i} \delta^{1 - z_i}$$

Given estimates $\vec{\phi}_t, \lambda_t, \delta_t$ at the t-th iteration, the $Q(\vec{\phi}, \lambda, \delta|\vec{\phi}_t, \lambda_t, \delta_t)$ function of EM is:

$$Q(\vec{\phi}, \lambda, \delta|\vec{\phi}_t, \lambda_t, \delta_t) = \sum_{\vec{z}} \sum_{\vec{g}} \Pr\{\vec{g}, \vec{z}|\vec{D}, \vec{\phi}_t, \lambda_t, \delta_t\} \log \Pr\{\vec{D}, \vec{g}, \vec{z}|\vec{\phi}, \lambda, \delta\}$$

$$= C + \sum_{\vec{z}} \sum_{\vec{g}} \prod_{i=1}^{n} \Pr\{g_i, z_i|\vec{D}_i, \vec{\phi}_t, \lambda_t, \delta_t\} \sum_{j} \log \Pr\{d_j|g_j, z_j, \lambda\} \Pr\{g_j|\vec{\phi}\} \Pr\{z_j|\delta\}$$

$$= C + \sum_{i=1}^{n} \sum_{z_i=0}^{1} \sum_{g_i} \Pr\{g_i, z_i|\vec{D}_i, \vec{\phi}_t, \lambda_t, \delta_t\} \log \Pr\{d_i|g_i, z_i, \lambda\} \Pr\{g_i|\vec{\phi}\} \Pr\{z_i|\delta\}$$

$$= C' + \sum_{i=1}^{n} \sum_{z_i=0}^{1} \sum_{g_i} \Pr\{g_i, z_i|\vec{D}_i, \vec{\phi}_t, \lambda_t, \delta_t\} \Big[d_i \log(\lambda) - (d_i + \psi) \log(\lambda z_i r_i \frac{m_i}{2} + \psi) +$$

$$g_{i,1} \log(\phi_1) + g_{i,2} \log(\phi_2) + g_{i,3} \log(\phi_3) + z_i \log(1 - \delta) + (1 - z_i) \log(\delta) \Big]$$

Thus

$$\frac{\partial Q}{\partial \phi_1} = \sum_{i=1}^{n} \sum_{z_i=0}^{1} \sum_{g_i} \Pr\{g_i, z_i | \vec{D}_i, \vec{\phi}_t, \lambda_t, \delta_t\} \frac{g_{i,1}}{\phi_1}$$

$$\frac{\partial Q}{\partial \phi_2} = \sum_{i=1}^{n} \sum_{z_i=0}^{1} \sum_{g_i} \Pr\{g_i, z_i | \vec{D}_i, \vec{\phi}_t, \lambda_t, \delta_t\} \frac{g_{i,2}}{\phi_2}$$

$$\frac{\partial Q}{\partial \phi_3} = \sum_{i=1}^{n} \sum_{z_i=0}^{1} \sum_{g_i} \Pr\{g_i, z_i | \vec{D}_i, \vec{\phi}_t, \lambda_t, \delta_t\} \frac{g_{i,3}}{\phi_3}$$

$$\frac{\partial Q}{\partial \delta} = \sum_{i=1}^{n} \sum_{z_i=0}^{1} \sum_{g_i} \Pr\{g_i, z_i | \vec{D}_i, \vec{\phi}_t, \lambda_t, \delta_t\} \left[\frac{1-z_i}{\delta} - \frac{z_i}{1-\delta} \right]$$

$$\frac{\partial Q}{\partial \lambda} = \sum_{i=1}^{n} \sum_{z_i=0}^{1} \sum_{g_i} \Pr\{g_i, z_i | \vec{D}_i, \vec{\phi}_t, \lambda_t, \delta_t\} \left[\frac{d_i}{\lambda} - \frac{z_i r_i \frac{m_i}{2}(d_i + \psi)}{\lambda z_i r_i \frac{m_i}{2} + \psi} \right]$$

and using a first-order Taylor expansion about the point $\lambda = \lambda_t$

$$\frac{\partial Q}{\partial \lambda} \approx \sum_{i=1}^{n} \sum_{z_i=0}^{1} \sum_{g_i} \Pr\{g_i, z_i | \vec{D}_i, \vec{\phi}_t, \lambda_t, \delta_t\} \left[\frac{d_i}{\lambda_t} - \frac{z_i r_i \frac{m_i}{2}(d_i + \psi)}{\lambda_t z_i r_i \frac{m_i}{2} + \psi} \right] + (\lambda - \lambda_t) \sum_{i=1}^{n} \sum_{z_i=0}^{1} \sum_{g_i} \Pr\{g_i, z_i | \vec{D}_i, \vec{\phi}_t, \lambda_t, \delta_t\} \left[\frac{(z_i r_i \frac{m_i}{2})^2 (d_i + \psi)}{(\lambda_t z_i r_i \frac{m_i}{2} + \psi)^2} - \frac{d_i}{\lambda_t^2} \right]$$

To calculate the updated parameter estimates we set each partial derivative equal to 0 and solve for ϕ_1, ϕ_2, ϕ_3, λ, and δ. Because of the constraint $\phi_1 + \phi_2 + \phi_3 = 1$ we introduce a Lagrange multiplier:

$$\rho = \sum_{i=1}^{n} \sum_{z_i=0}^{1} \sum_{g_i} \Pr\{g_i, z_i | \vec{D}_i, \vec{\phi}_t, \lambda_t, \delta_t\} (g_{i,1} + g_{i,2} + g_{i,3}) = 2n$$

Thus

$$\phi_{1(t+1)} = \frac{1}{2n} \sum_{i=1}^{n} \sum_{z_i=0}^{1} \sum_{g_i} \Pr\{g_i, z_i | \vec{D}_i, \vec{\phi}_t, \lambda_t, \delta_t\} g_{i,1}$$

$$\phi_{2(t+1)} = \frac{1}{2n} \sum_{i=1}^{n} \sum_{z_i=0}^{1} \sum_{g_i} \Pr\{g_i, z_i | \vec{D}_i, \vec{\phi}_t, \lambda_t, \delta_t\} g_{i,2}$$

$$\phi_{3(t+1)} = \frac{1}{2n} \sum_{i=1}^{n} \sum_{z_i=0}^{1} \sum_{g_i} \Pr\{g_i, z_i | \vec{D}_i, \vec{\phi}_t, \lambda_t, \delta_t\} g_{i,3}$$

and

$$\delta_{t+1} = \frac{1}{n} \sum_{i=1}^{n} \sum_{z_i=0}^{1} \sum_{g_i} \Pr\{g_i, z_i | \vec{D}_i, \vec{\phi}_t, \lambda_t, \delta_t\}(1 - z_i)$$

$$\lambda_{t+1} = \lambda_t - \frac{\sum_{i=1}^{n} \sum_{z_i=0}^{1} \sum_{g_i} \left[\frac{d_i}{\lambda_t} - \frac{z_i r_i \frac{m_i}{2}(d_i + \psi)}{z_i r_i \frac{m_i}{2}\lambda_t + \psi} \right] \Pr\{g_i, z_i | \vec{D}_i, \vec{\phi}_t, \lambda_t, \delta_t\}}{\sum_{i=1}^{n} \sum_{z_i=0}^{1} \sum_{g_i} \left[\frac{(z_i r_i \frac{m_i}{2})^2 (d_i + \psi)}{(z_i r_i \frac{m_i}{2}\lambda_t + \psi)^2} - \frac{d_i}{\lambda_t^2} \right] \Pr\{g_i, z_i | \vec{D}_i, \vec{\phi}_t, \lambda_t, \delta_t\}}$$

thus

$$\phi_{1(t+1)} = \frac{1}{2n} \sum_{i=1}^{n} \frac{1}{C_i} \sum_{z_i=0}^{1} \sum_{g_i} g_{i,1} \Pr\{\vec{D}_i, g_i, z_i | \vec{\phi}_t, \lambda_t, \delta_t\} \tag{1}$$

$$\phi_{2(t+1)} = \frac{1}{2n} \sum_{i=1}^{n} \frac{1}{C_i} \sum_{z_i=0}^{1} \sum_{g_i} g_{i,2} \Pr\{\vec{D}_i, g_i, z_i | \vec{\phi}_t, \lambda_t, \delta_t\} \tag{2}$$

$$\phi_{3(t+1)} = \frac{1}{2n} \sum_{i=1}^{n} \frac{1}{C_i} \sum_{z_i=0}^{1} \sum_{g_i} g_{i,3} \Pr\{\vec{D}_i, g_i, z_i | \vec{\phi}_t, \lambda_t, \delta_t\} \tag{3}$$

$$\delta_{t+1} = \frac{1}{n} \sum_{i=1}^{n} \frac{1}{C_i} \sum_{z_i=0}^{1} \sum_{g_i} (1 - z_i) \Pr\{\vec{D}_i, g_i, z_i | \vec{\phi}_t, \lambda_t, \delta_t\} \tag{4}$$

$$\lambda_{t+1} = \lambda_t - \frac{\sum_{i=1}^{n} \frac{1}{C_i} \sum_{z_i=0}^{1} \sum_{g_i} \left[\frac{d_i}{\lambda_t} - \frac{z_i r_i \frac{m_i}{2}(d_i + \psi)}{z_i r_i \frac{m_i}{2}\lambda_t + \psi} \right] \Pr\{\vec{D}_i, g_i, z_i | \vec{\phi}_t, \lambda_t, \delta_t\}}{\sum_{i=1}^{n} \frac{1}{C_i} \sum_{z_i=0}^{1} \sum_{g_i} \left[\frac{(z_i r_i \frac{m_i}{2})^2 (d_i + \psi)}{(z_i r_i \frac{m_i}{2}\lambda_t + \psi)^2} - \frac{d_i}{\lambda_t^2} \right] \Pr\{\vec{D}_i, g_i, z_i | \vec{\phi}_t, \lambda_t, \delta_t\}} \tag{5}$$

where

$$\Pr\{\vec{D}_i, g_i, z_i | \vec{\phi}_t, \lambda_t, \delta_t\} = \Pr\{\vec{D}_i | g_i, d_i\} \Pr\{d_i | g_i, z_i, \lambda_t\} \Pr\{g_i | \vec{\phi}_t\} \Pr\{z_i | \delta_t\}$$

and

$$C_i = \sum_{z_i=0}^{1} \sum_{g_i} \Pr\{\vec{D}_i, g_i, z_i | \vec{\phi}_t, \lambda_t, \delta_t\}$$

References

[1] Li H. Mathematical notes on SAMtools algorithms. www.broadinstitute.org/gatk/media/docs/Samtools.pdf
