# Supplementary material for: GBStools: A Statistical Method for Estimating Allelic Dropout in Reduced Representation Sequencing Data
Source: PLoS Genet. 2016 Feb 1;12(2):e1005631. doi: 10.1371/journal.pgen.1005631 (PMC4734769; doi:10.1371/journal.pgen.1005631)
Supplement: S1 Fig — A. The 10.38 kb bioanalyzer marker is used as an internal standard for DNA quantification, but if the sample to be quantified is overloaded or bleeds into the 10kb region of the electropherogram, the bioanalyzer software may overestimate the concentration of the marker, as shown here. This leads to incorrect estimation of the sample concentration. B. We reanalyzed bioanalyzer data from the first batch of 24 Argentine samples we sequenced, and plotted the total number of reads from each sample vs the quantity of DNA added to the final library pool based on the recalculated DNA concentration. (PDF) [file pgen.1005631.s002.pdf]

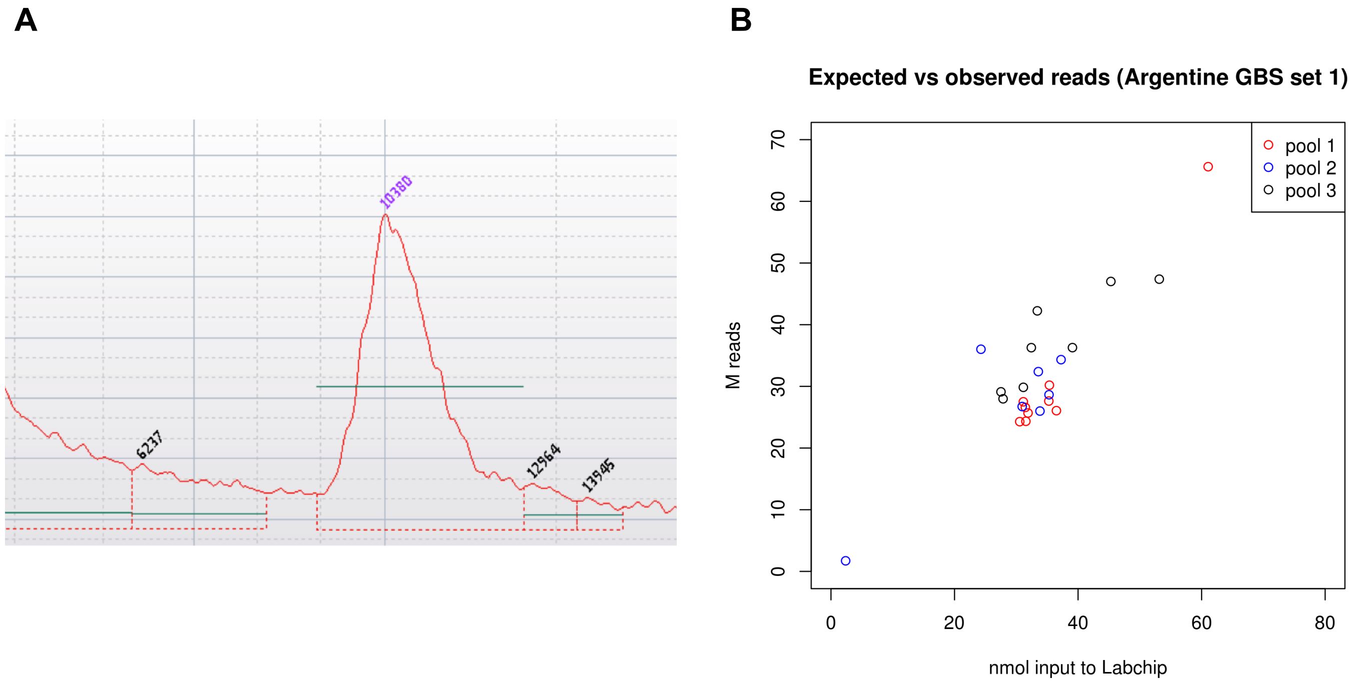

S1 Fig. Post-hoc re-quantification of Argentine GBS libraries. A. The 10.38 kb bioanalyzer marker is used as an internal standard for DNA quantification, but if the sample to be quantified is overloaded or bleeds into the 10kb region of the electropherogram, the bioanalyzer software may overestimate the concentration of the marker, as shown here. This leads to incorrect estimation of the sample concentration. **B.** We reanalyzed bioanalyzer data from the first batch of 24 Argentine samples we sequenced, and plotted the total number of reads from each sample vs the quantity of DNA added to the final library pool based on the recalculated DNA concentration.
