# Supplementary material for: GBStools: A Statistical Method for Estimating Allelic Dropout in Reduced Representation Sequencing Data
Source: PLoS Genet. 2016 Feb 1;12(2):e1005631. doi: 10.1371/journal.pgen.1005631 (PMC4734769; doi:10.1371/journal.pgen.1005631)
Supplement: S2 Fig — A. The target region was defined to be the union of simulated digest fragments between 400–700 bp (200–600 bp for the Argentine samples) that had ≥ 3X mean coverage per sample and where ≥ 10% of mate pairs were mapped to the restriction sites at the end of the fragment. B. Bioinformatics flowchart. SNP were called in the target region with GATK. Variant quality score recalibration (VQSR) was performed. Sites passing the basic filters had: mapping quality ≥ 57, SNP quality ≥ 30, coverage ≥ 8X in all samples (HapMap samples) or coverage ≥ 8X in ≥ 40/63 samples (Argentine samples), and position outside the 1000 Genomes Project callability mask. Sites failing the 1000 Genomes filter had > 10% spanning reads mapped to known polymorphic restriction site (allele frequency > 0.01). Sites failing the GBStools filter had: Non-cut allele frequency estimate > 0.05, or likelihood ratio > 2.71 (p < 0.05). Additional details on the filters are included in the methods section. (PDF) [file pgen.1005631.s003.pdf]

A Determination of GBS target region:

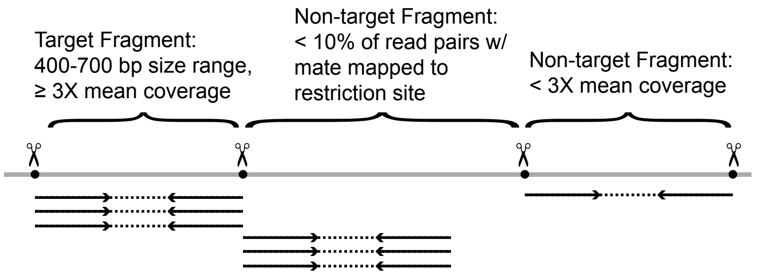

B

Sequencing: Illumina HiSeq 2000

Mapping

Mapping to GRCh37:	BWA
Quality score recalibration	GATK
Local realignment	GATK
Add restriction site tag to aligned reads	GBStools

Calling

Make bed file of GBS target sites	custom scripts
Call genotypes at GBS target sites	GATK
Calculate coverage normalization factors	GBStools
Test for restriction site variation	GBStools

HapMap Samples

Filtering	All sites	Segregating sites
GBS target (autosomes)	127,658,223	549,886
VQSR	127,591,607	483,381
Basic filters	32,446,886	118,122
1000 Genomes variant restriction sites	30,033,800	108,349
GBStools	29,246,187	99,534

Argentine Samples

Filtering	All sites	Segregating sites
GBS target (autosomes)	177,276,749	1,013,785
VQSR	177,027,689	767,427
Basic filters	16,736,745	69,846
1000 Genomes variant restriction sites	14,992,650	61,592
GBStools	12,747,025	42,598

Argentine + HGDP + 1000 Genomes Project samples (filters for PCA)

Filtering	Segregating sites
GBS target (autosomes)	1,977,487
Site was called in each data set	715,082
Missingness filter	59,004
Basic filters	54,422
Thinned by LD	45,630

S2 Fig. GBS SNP filtering. A. The target region was defined to be the union of simulated digest fragments between 400-700 bp (200-600 bp for the Argentine samples) that had ≥ 3X mean coverage per sample and where ≥ 10% of mate pairs were mapped to the restriction sites at the end of the fragment. **B.** Bioinformatics flowchart. SNPs were called in the target region with GATK. Variant quality score recalibration (VQSR) was performed. Sites passing the basic filters had: mapping quality ≥ 57, SNP quality ≥ 30, coverage ≥ 8X in all samples (HapMap samples) or coverage ≥ 8X in ≥ 40/63 samples (Argentine samples), and position outside the 1000 Genomes Project callability mask. Sites failing the 1000 Genomes filter had > 10% spanning reads mapped to known polymorphic restriction site (allele frequency > 0.01). Sites failing the GBStools filter had: Non-cut allele frequency estimate > 0.05, or likelihood ratio > 2.71 ($p < 0.05$). Additional details on the filters are included in the online methods.
