# Supplementary material for: GBStools: A Statistical Method for Estimating Allelic Dropout in Reduced Representation Sequencing Data
Source: PLoS Genet. 2016 Feb 1;12(2):e1005631. doi: 10.1371/journal.pgen.1005631 (PMC4734769; doi:10.1371/journal.pgen.1005631)
Supplement: S3 Fig — A. Distribution of mean GBS coverage across eight HapMap samples for all sites in the genome, and for sites in the target region. B. Distribution of mean GBS coverage across the 63 Argentine samples that had ≥ 30% of reads mapped to restriction sites. (PDF) [file pgen.1005631.s004.pdf]

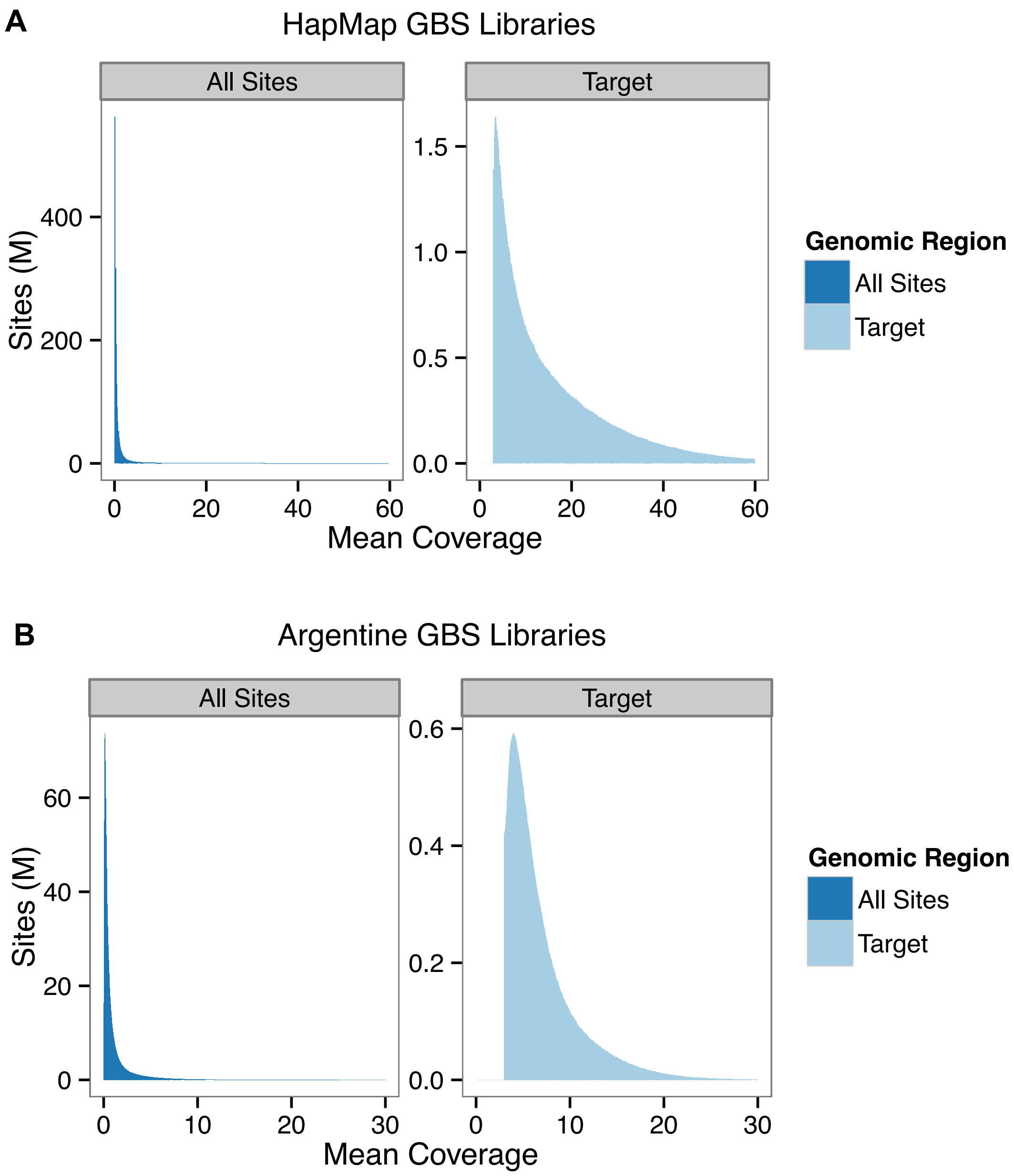

S3 Fig. Coverage distributions for HapMap and Argentine GBS libraries. A. Distribution of mean GBS coverage across eight HapMap samples for all sites in the genome, and for sites in the target region. **B.** Distribution of mean GBS coverage across the 63 Argentine samples that had $\geq$ 30% of reads mapped to restriction sites.
