# Supplementary material for: GBStools: A Statistical Method for Estimating Allelic Dropout in Reduced Representation Sequencing Data
Source: PLoS Genet. 2016 Feb 1;12(2):e1005631. doi: 10.1371/journal.pgen.1005631 (PMC4734769; doi:10.1371/journal.pgen.1005631)
Supplement: S4 Fig — A. Double-digest GBS genotype data (5×10^5^ SNPs) were simulated for 100 diploid individuals under a neutral coalescent model with population mutation rates between 1×10^−3^ and 2×10^−2^, and the genotype error rates plotted vs scaled mutation rate after removing sites with > 10% missing genotypes. The error rates after removing sites with a GBStools likelihood ratio > 2.71 are also shown for the same data with 40X mean coverage. B. Site frequency spectrum for restriction site polymorphisms for data simulated under a population mutation rate of 1×10^−3^. Samples that carried two non-cut restriction site alleles were considered missing. C. The number of SNP genotyping errors is shown for each frequency class in B. D. The non-normalized SNP site frequency spectra for the same data as in A, represented as a subsample of size 50. E. The normalized site frequency spectra corresponding to those shown in D. (PDF) [file pgen.1005631.s005.pdf]

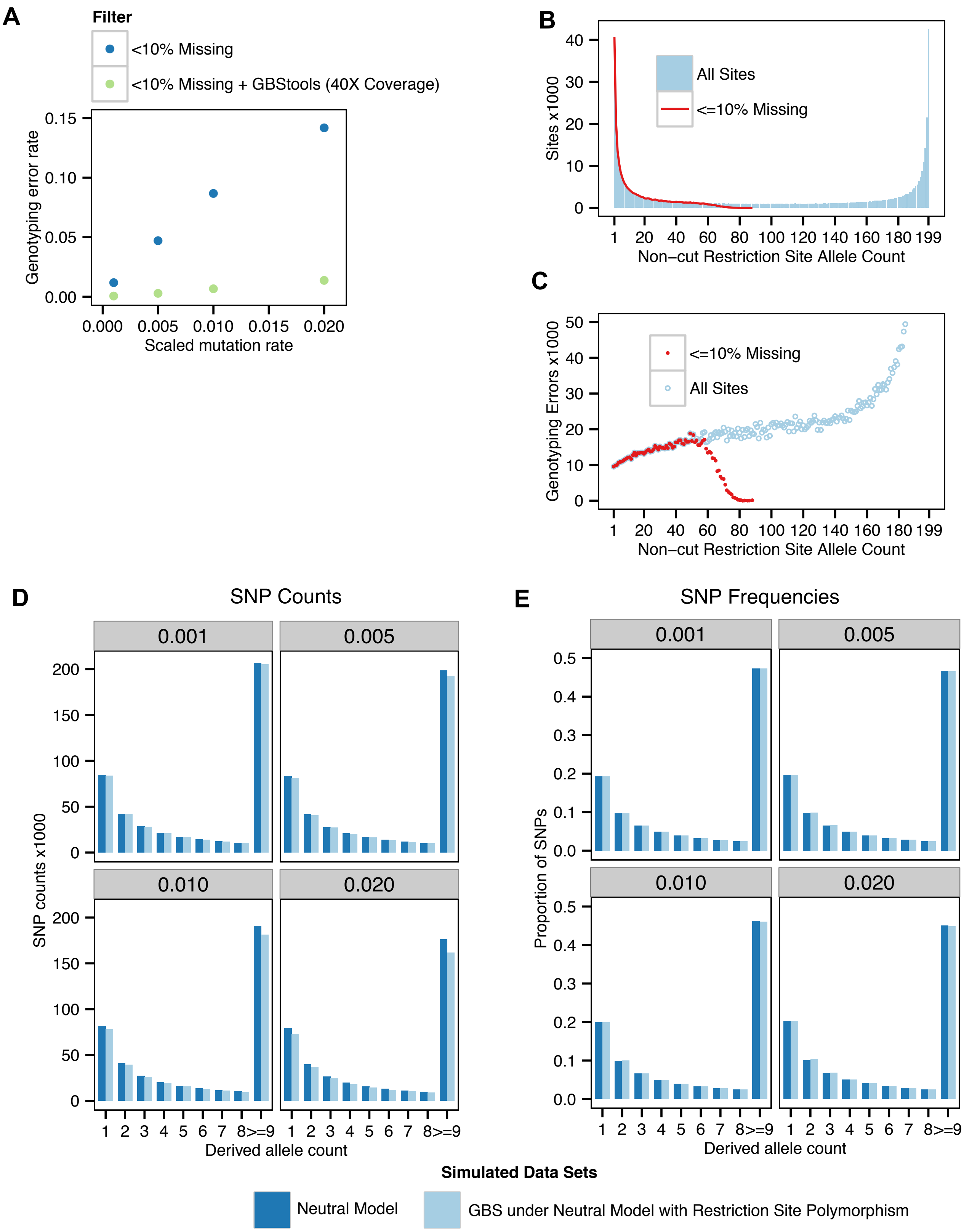

S4 Fig. Effect of restriction site polymorphisms on genotyping accuracy and site frequency spectrum estimation in simulated GBS data. A. Double-digest GBS genotype data (5×10^5 SNPs) were simulated for 100 diploid individuals under a neutral coalescent model with population mutation rates between 1×10^{-3} and 2×10^{-2}, and the genotype error rates plotted vs scaled mutation rate after removing sites with > 10% missing genotypes. The error rates after removing sites with a GBStools likelihood ratio > 2.71 are also shown for the same data with 40X mean coverage. B. Site frequency spectrum for restriction site polymorphisms for data simulated under a population mutation rate of 1×10^{-3}. Samples that carried two non-cut restriction site alleles were considered missing. C. The number of SNP genotyping errors is shown for each frequency class in B. D. The non-normalized SNP site frequency spectra for the same data as in A, represented as a subsample of size 50. E. The normalized site frequency spectra corresponding to those shown in D.
