# Supplementary material for: GBStools: A Statistical Method for Estimating Allelic Dropout in Reduced Representation Sequencing Data
Source: PLoS Genet. 2016 Feb 1;12(2):e1005631. doi: 10.1371/journal.pgen.1005631 (PMC4734769; doi:10.1371/journal.pgen.1005631)
Supplement: S5 Fig — Target-region restriction sites were binned by mean coverage (bin width = 0.5), and dispersion index for coverage across samples (bin width = 0.1). The number of sites per bin is indicated by shade. Coverage was normalized at each site to account for variation in total read number per sample (methods). In estimating allele frequencies, GBStools used a fixed value for the dispersion index that was a linear function of the mean site coverage. (PDF) [file pgen.1005631.s006.pdf]

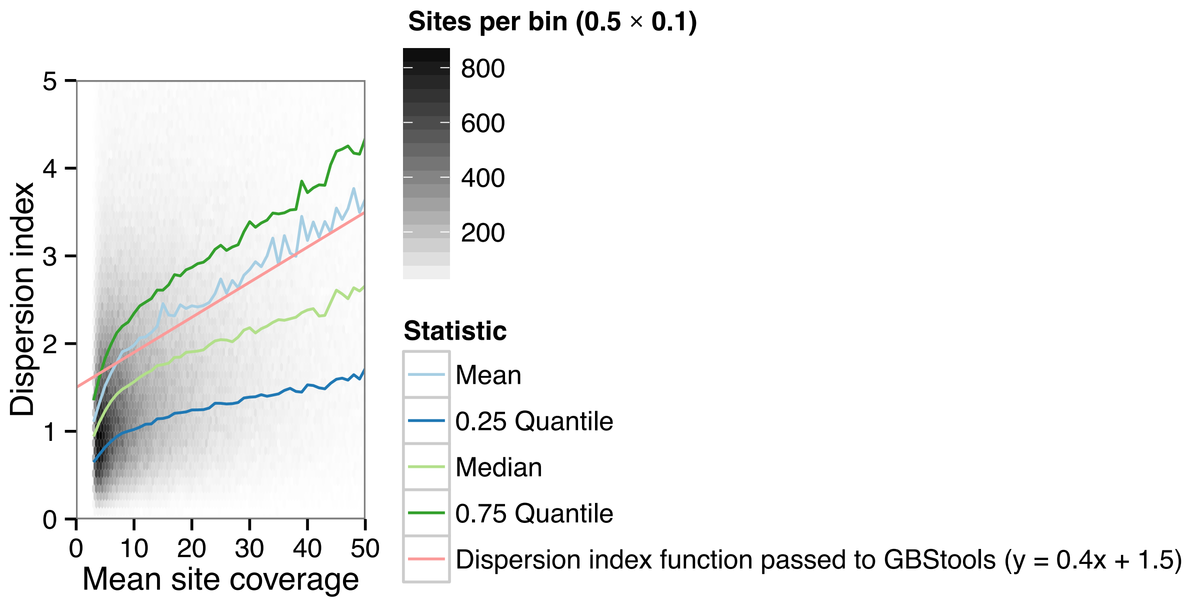

S5 Fig. Dispersion of read coverage across samples at SNPs in HapMap GBS data set. Target-region restriction sites were binned by mean coverage (bin width = 0.5), and dispersion index for coverage across samples (bin width = 0.1). The number of sites per bin is indicated by shade. Coverage was normalized at each site to account for variation in total read number per sample (methods). In estimating allele frequencies, GBStools used a fixed value for the dispersion index that was a linear function of the mean site coverage.
