# Supplementary material for: GBStools: A Statistical Method for Estimating Allelic Dropout in Reduced Representation Sequencing Data
Source: PLoS Genet. 2016 Feb 1;12(2):e1005631. doi: 10.1371/journal.pgen.1005631 (PMC4734769; doi:10.1371/journal.pgen.1005631)
Supplement: S6 Fig — A. The GBStools likelihood ratio statistic was calculated for SNPs in a simulated GBS data set that originated from a monomorphic restriction site, with either 8 or 100 samples, and with coverage of 10, 20, 30, 40, 50, 100, or 200X (methods). Each plot compares the quantiles of the likelihood ratio statistic to the quantiles of the expected null distribution, a one-half chi-squared distribution with one degree of freedom. B. Q-Q plots for sites in the simulated data where 4/16 or 4/200 chromosomes carried the non-cut restriction site allele. The power of the likelihood ratio test was calculated with a critical value of 2.71 (p < 0.05). (PDF) [file pgen.1005631.s007.pdf]

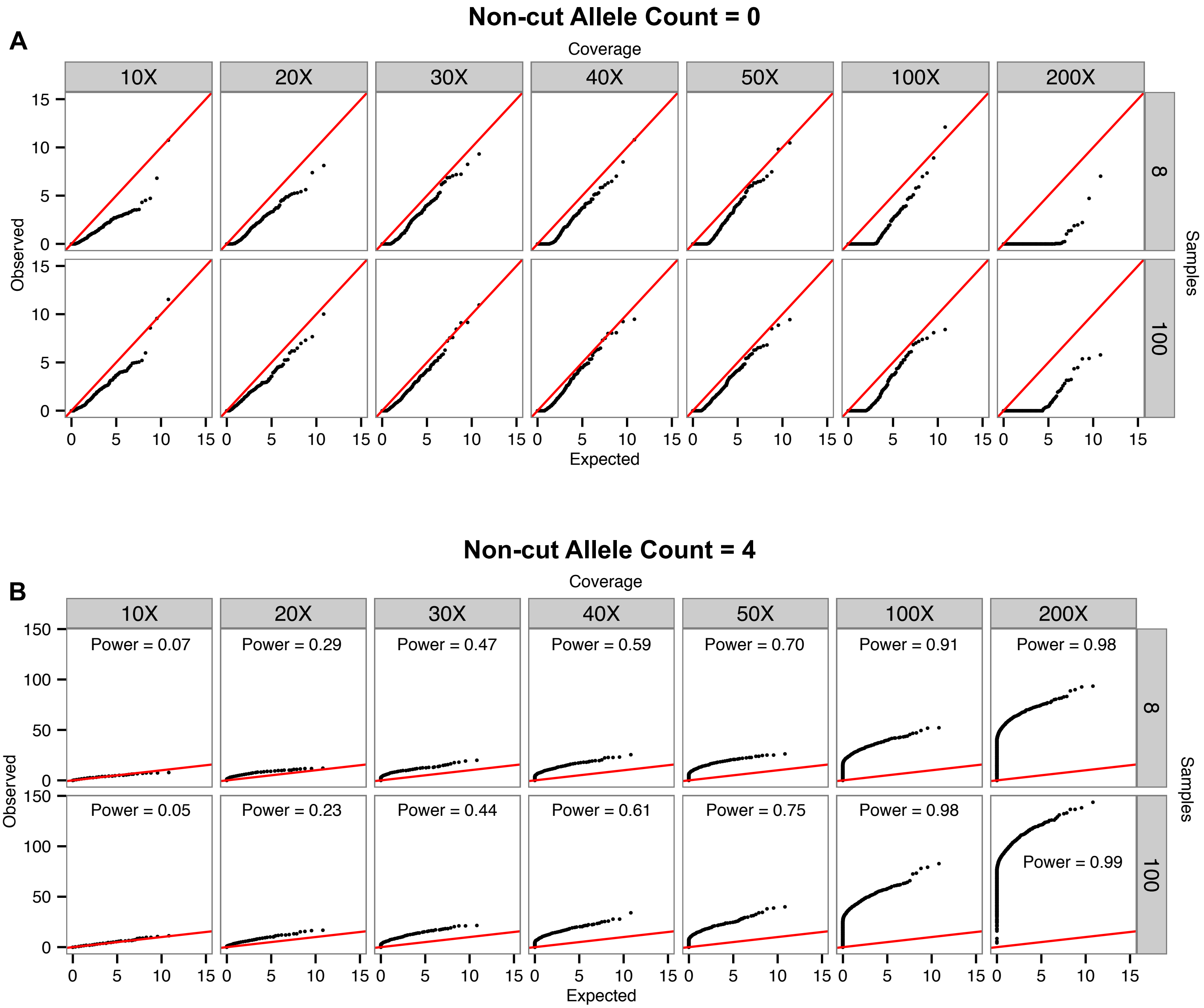

S6 Fig. Quantile-quantile plots for restriction site polymorphism likelihood ratio statistic. A. The GBStools likelihood ratio statistic was calculated for SNPs in a simulated GBS data set that originated from a monomorphic restriction site, with either 8 or 100 samples, and with coverage of 10, 20, 30, 40, 50, 100, or 200X (methods). Each plot compares the quantiles of the likelihood ratio statistic to the quantiles of the expected null distribution, a one-half chi-squared distribution with one degree of freedom. **B.** Q-Q plots for sites in the simulated data where 4/16 or 4/200 chromosomes carried the non-cut restriction site allele. The power of the likelihood ratio test was calculated with a critical value of 2.71 ($p < 0.05$).
