# Supplementary material for: GBStools: A Statistical Method for Estimating Allelic Dropout in Reduced Representation Sequencing Data
Source: PLoS Genet. 2016 Feb 1;12(2):e1005631. doi: 10.1371/journal.pgen.1005631 (PMC4734769; doi:10.1371/journal.pgen.1005631)
Supplement: S7 Fig — A. Estimates for the coverage parameter, λ, for the simulated GBS data set. B. Estimates for the non-cutter restriction site allele frequency parameter, ϕ3, for the simulated GBS data set. (PDF) [file pgen.1005631.s008.pdf]

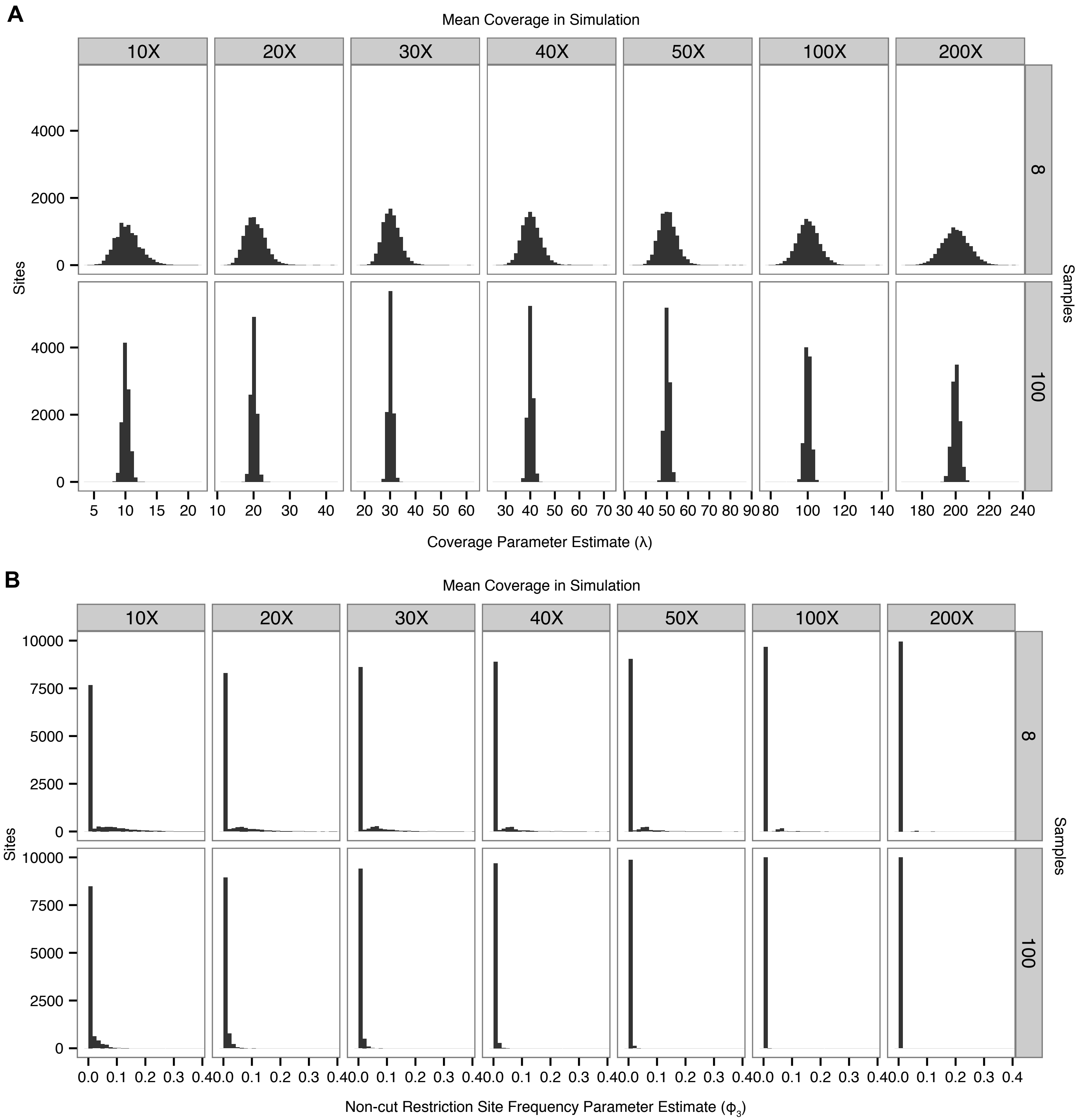

S7 Fig. Distributions of GBStools maximum likelihood parameter estimates for simulated GBS data with non-cut restriction site allele frequency = 0 (null model). A. Estimates for the coverage parameter, λ, for the simulated GBS data set. **B.** Estimates for the non-cut restriction site allele frequency parameter, ϕ_3, for the simulated GBS data set.
