# Supplementary material for: GBStools: A Statistical Method for Estimating Allelic Dropout in Reduced Representation Sequencing Data
Source: PLoS Genet. 2016 Feb 1;12(2):e1005631. doi: 10.1371/journal.pgen.1005631 (PMC4734769; doi:10.1371/journal.pgen.1005631)
Supplement: S8 Fig — The test statistic was calculated as described in S1 Appendix for 333,058 autosomal SNPs in the HapMap GBS data set that had < 25% missing data. Each plot represents a group of SNPs that were sequenced to a different mean coverage, and within each group the test statistic is plotted versus the number of non-cut restriction site alleles present at the SNP. The blue lines represent the p = 0.05 critical value from the theoretical null distribution, the one-half chi-squared distribution with one degree of freedom. The red lines represent the empirical p = 0.05 critical value. (PDF) [file pgen.1005631.s009.pdf]

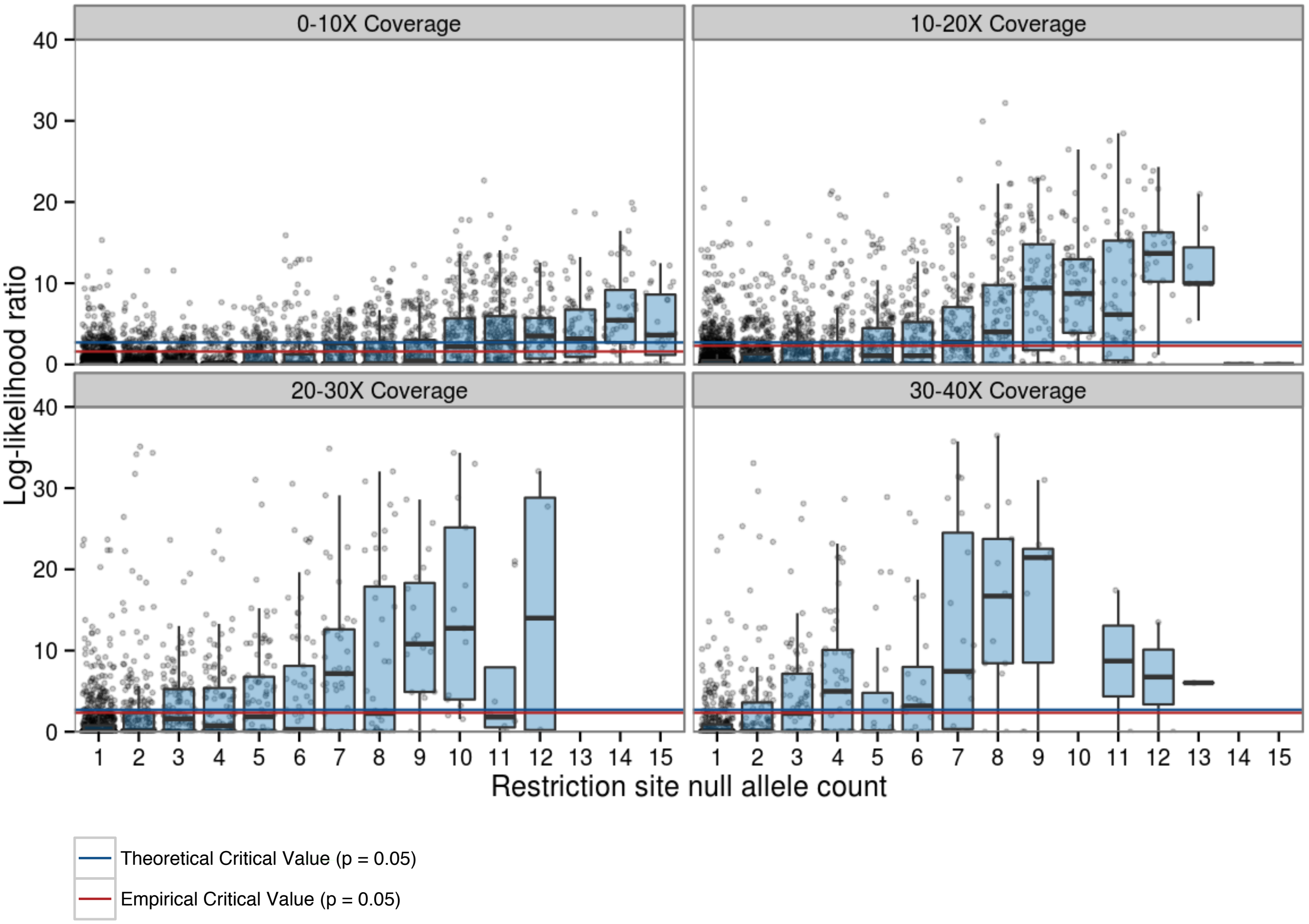

S8 Fig. Distributions of likelihood ratio statistic calculated from GBS read data at known polymorphic restriction sites. The test statistic was calculated as described in S1 Appendix for 333,058 autosomal SNPs in the HapMap GBS data set that had < 25% missing data. Each plot represents a group of SNPs that were sequenced to a different mean coverage, and within each group the test statistic is plotted versus the number of non-cut restriction site alleles present at the SNP. The blue lines represent the $p = 0.05$ critical value from the theoretical null distribution, the one-half chi-squared distribution with one degree of freedom. The red lines represent the empirical $p = 0.05$ critical value.
