# Supplementary material for: GBStools: A Statistical Method for Estimating Allelic Dropout in Reduced Representation Sequencing Data
Source: PLoS Genet. 2016 Feb 1;12(2):e1005631. doi: 10.1371/journal.pgen.1005631 (PMC4734769; doi:10.1371/journal.pgen.1005631)
Supplement: S1 Table — Populations are annotated as YRI (Yoruban from Nigeria), MXL (Mexican American from Los Angeles), ASW (African-American from South West USA) and MKK (Maassai from Kenya). Genomes were sequences with either Complete Genomics or ABI SOLiD technologies; read pairs are given per million (M), and coverage is given in fold-coverage. The target region is defined to be the union of simulated digest fragments between 400–700 bp that had ≥ 3X mean coverage per sample and where ≥ 10% of mate pairs were mapped to restriction sites at the end of the fragment. (PDF) [file pgen.1005631.s010.pdf]

						Mapped to restriction site:			
**Sample**	**Population**	**WGS data**	**Read pairs (M)**	**Mapped pairs (%)**	**On target pairs (%)**	**0 of 2 in pair (%)**	**1 of 2 In pair (%)**	**2 of 2 In pair (%)**	**Coverage in target**
NA18505	YRI	Complete Genomics	18.2	97.5	73.3	7.4	23.0	69.6	18.9
NA18508	YRI	Complete Genomics	21.1	97.6	55.0	12.5	33.5	54.0	16.2
NA19648	MXL	Complete Genomics	22.8	97.3	57.6	14.2	34.7	51.1	18.5
NA19704	ASW	Complete Genomics	13.3	97.3	63.0	12.8	32.6	54.5	12.0
NA19730	MXL	SOLiD	13.5	98.1	67.4	10.5	29.6	59.9	13.8
NA19836	ASW	SOLiD	17.9	97.5	55.5	11.8	30.9	57.3	14.0
NA21732	MKK	Complete Genomics	19.8	98.0	76.1	6.5	24.7	68.8	23.0
NA21733	MKK	Complete Genomics	20.0	97.4	62.6	9.3	27.2	63.5	17.6
Mean	—	—	18.3	97.6	63.8	10.6	29.5	59.8	16.7

S1 Table. Read mapping results for HapMap individuals. Populations are annotated as YRI (Yoruban from Nigeria), MXL (Mexican American from Los Angeles), ASW (African-American from South West USA) and MKK (Maassai from Kenya). Genomes were sequences with either Complete Genomics or ABI SOLiD technologies; read pairs are given per million (M), and coverage is given in fold-coverage. The target region is defined to be the union of simulated digest fragments between 400-700 bp that had ≥ 3X mean coverage per sample and where ≥ 10% of mate pairs were mapped to restriction sites at the end of the fragment.
