# Supplementary material for: GBStools: A Statistical Method for Estimating Allelic Dropout in Reduced Representation Sequencing Data
Source: PLoS Genet. 2016 Feb 1;12(2):e1005631. doi: 10.1371/journal.pgen.1005631 (PMC4734769; doi:10.1371/journal.pgen.1005631)
Supplement: S2 Table — A-E. HapMap samples (SNPs called with GATK UnifiedGenotyper). Sites are grouped by the Complete Genomics genotype call: homozygous reference (0/0), heterozygous (0/1), or homozygous non-reference (1/1). For each group, the proportion called 0/0, 0/1, or 1/1 by GBS is shown, followed by the total number of sites in the group. F-G. HapMap samples (SNPs called with GATK HaplotypeCaller). H-K. Argentine samples (SNPs called with GATK UnifiedGenotyper). Sites are grouped by the Illumina Exome Array genotype call. A detailed description of the filters is provided in the methods section. (PDF) [file pgen.1005631.s011.pdf]

a

Hapmap GBS calls (basic filters)

GBS calls:		0/0	0/1	1/1	Totals
Complete Genomics Calls:	0/0	0.9997	0.0003	0.0000	57,345
	0/1	0.0168	0.9797	0.0035	29,388
	1/1	0.0002	0.0004	0.9994	14,450

b

Hapmap GBS calls (basic + GBStools filters)

GBS Calls:		0/0	0/1	1/1	Totals
Complete Genomics Calls:	0/0	0.9997	0.0003	NA	52,091
	0/1	0.0132	0.9847	0.0021	26,971
	1/1	0.0002	0.0004	0.9994	12,922

c

Hapmap GBS calls (basic + 1000 Genomes filters)

GBS Calls:		0/0	0/1	1/1	Totals
Complete Genomics Calls:	0/0	0.9997	0.0003	0.0000	52,167
	0/1	0.0084	0.9903	0.0014	26,771
	1/1	0.0001	0.0004	0.9994	13,199

d

Hapmap GBS calls (basic + 1000 Genomes + GBStools filters)

GBS Calls:		0/0	0/1	1/1	Totals
Complete Genomics Calls:	0/0	0.9997	0.0003	NA	47,947
	0/1	0.0076	0.9913	0.0010	24,857
	1/1	0.0002	0.0004	0.9994	11,954

e

Hapmap GBS calls (basic + Hardy-Weinberg filters)

GBS Calls:		0/0	0/1	1/1	Totals
Complete Genomics Calls:	0/0	0.9997	0.0003	0.0000	56,841
	0/1	0.0166	0.9800	0.0033	29,215
	1/1	0.0002	0.0004	0.9994	14,037

f

Hapmap HaplotypeCaller GBS calls (basic filters)

GBS Calls:		0/0	0/1	1/1	Totals
Complete Genomics Calls:	0/0	0.9999	0.0001	NA	53,301
	0/1	0.0476	0.9488	0.0035	27,939
	1/1	0.0105	0.0005	0.9890	13,716

g

Hapmap HaplotypeCaller GBS calls (basic + RGQ filters)

GBS Calls:		0/0	0/1	1/1	Totals
Complete Genomics Calls:	0/0	0.9999	0.0001	NA	52,632
	0/1	0.0245	0.9719	0.0036	27,276
	1/1	0.0001	0.0005	0.9994	13,574

h

Argentine GBS calls (basic filters)

GBS Calls:		0/0	0/1	1/1	Totals
Illumina Exome Array Calls	0/0	0.9999	0.0001	NA	2,547
	0/1	0.0061	0.9930	0.0009	82
	1/1	0.0149	0.0022	0.9829	52

i

Argentine GBS calls (basic + GBStools filters)

GBS Calls:		0/0	0/1	1/1	Totals
Illumina Exome Array Calls:	0/0	0.9999	0.0001	NA	1,478
	0/1	0.0014	0.9971	0.0014	54
	1/1	NA	0.0023	0.9977	34

j

Argentine GBS calls (basic + 1000 Genomes filters)

GBS Calls:		0/0	0/1	1/1	Totals
Illumina Exome Array Calls:	0/0	0.9999	0.0001	NA	2,291
	0/1	0.0026	0.9964	0.0010	74
	1/1	0.0170	0.0026	0.9804	45

k

Argentine GBS calls (basic + 1000 Genomes + GBStools filters)

GBS Calls:		0/0	0/1	1/1	Totals
Illumina Exome Array Calls:	0/0	0.9999	0.0001	NA	1,344
	0/1	0.0015	0.9969	0.0015	50
	1/1	NA	0.0025	0.9975	31

k

Argentine GBS calls (basic + Hardy-Weinberg filters)

GBS Calls:		0/0	0/1	1/1	Totals
Illumina Exome Array Calls:	0/0	0.9999	0.0001	NA	2,534
	0/1	0.0020	0.9970	0.0010	78
	1/1	0.0156	0.0023	0.9820	49

S2 Table. Genotype concordance tables for HapMap and Argentine individuals with different combinations of filters. A-E. HapMap samples (SNPs called with GATK UnifiedGenotyper). Sites are grouped by the Complete Genomics genotype call: homozygous reference (0/0), heterozygous (0/1), or homozygous non-reference (1/1). For each group, the proportion called 0/0, 0/1, or 1/1 by GBS is shown, followed by the total number of sites in the group. **F-G.** HapMap samples (SNPs called with GATK HaplotypeCaller). **H-K.** Argentine samples (SNPs called with GATK UnifiedGenotyper). Sites are grouped by the Illumina Exome Array genotype call. A detailed description of the filters is provided in the methods section.
