# Supplementary material for: GBStools: A Statistical Method for Estimating Allelic Dropout in Reduced Representation Sequencing Data
Source: PLoS Genet. 2016 Feb 1;12(2):e1005631. doi: 10.1371/journal.pgen.1005631 (PMC4734769; doi:10.1371/journal.pgen.1005631)
Supplement: S3 Table — The GBStools likelihood ratio test or Hardy-Weinberg equilibrium exact test p-values were used as classifiers of incorrect vs correct heterozygous genotype calls for simulated and empirical data, and sensitivity and specificity were calculated for various classifier thresholds (Fig 4A). There were 2×10^5^ sites in each simulated data set (θ = 4Neμ = 1×10^−3^), which included sites with and without restriction site variation. A 90% call rate filter was applied to the simulated data before calculating error rates, and "basic filters" (S2 Fig) were first applied to the HapMap and Argentine data. For reference, an uninformative (random) classifier has AUC = 0.5 and a perfect classifier has AUC = 1.0. The total number of correctly called genotypes (non-error genotypes) and incorrectly called (error) genotypes are shown in the rightmost columns. (PDF) [file pgen.1005631.s012.pdf]

Data Set	Samples	Classifier	Coverage	AUC	Non-error Genotypes	Error Genotypes
Simulation	8	GBStools	10X	0.59	470110	5660
Simulation	8	GBStools	20X	0.73	470110	5660
Simulation	8	GBStools	30X	0.79	470110	5660
Simulation	8	GBStools	40X	0.83	470110	5660
Simulation	8	GBStools	50X	0.87	470110	5660
Simulation	8	GBStools	100X	0.93	470110	5660
Simulation	8	Hardy-Weinberg	NA	0.50	470110	5660
Simulation	30	GBStools	10X	0.59	1259275	17393
Simulation	30	GBStools	20X	0.81	1259275	17393
Simulation	30	GBStools	30X	0.89	1259275	17393
Simulation	30	GBStools	40X	0.93	1259275	17393
Simulation	30	GBStools	50X	0.95	1259275	17393
Simulation	30	GBStools	100X	0.99	1259275	17393
Simulation	30	Hardy-Weinberg	NA	0.43	1259275	17393
Simulation	100	GBStools	10X	0.64	3316599	41129
Simulation	100	GBStools	20X	0.84	3316599	41129
Simulation	100	GBStools	30X	0.91	3316599	41129
Simulation	100	GBStools	40X	0.96	3316599	41129
Simulation	100	GBStools	50X	0.98	3316599	41129
Simulation	100	GBStools	100X	0.99	3316599	41129
Simulation	100	Hardy-Weinberg	NA	0.42	3316599	41129
Simulation	500	GBStools	10X	0.70	13017240	161283
Simulation	500	GBStools	20X	0.83	13017240	161283
Simulation	500	GBStools	30X	0.92	13017240	161283
Simulation	500	GBStools	40X	0.98	13017240	161283
Simulation	500	GBStools	50X	0.99	13017240	161283
Simulation	500	GBStools	100X	0.99	13017240	161283
Simulation	500	Hardy-Weinberg	NA	0.39	13017240	161283
HapMap	8	GBStools	5-15X	0.52	7592	162
HapMap	8	GBStools	15-25X	0.59	57668	1252
HapMap	8	GBStools	25-35X	0.66	50397	1072
HapMap	8	GBStools	35-45X	0.69	28950	632
HapMap	8	GBStools	45-55X	0.74	14921	270
HapMap	8	Hardy-Weinberg	NA	0.48	172747	3578
Argentine	63	GBStools	5-15X	0.81	1045	15
Argentine	63	Hardy-Weinberg	NA	0.75	2124	15

S3 Table. Area under curve (AUC) for response operator characteristic (ROC) curves. The GBStools likelihood ratio test or Hardy-Weinberg equilibrium exact test p-values were used as classifiers of incorrect vs correct heterozygous genotype calls for simulated and empirical data, and sensitivity and specificity were calculated for various classifier thresholds (Fig. 4A). There were 2×10^5 sites in each simulated data set ($\theta = 4N_e\mu = 1\times10^{-3}$), which included sites with and without restriction site variation. A 90 % call rate filter was applied to the simulated data before calculating error rates, and "basic filters" (S2 Fig.) were first applied to the HapMap and Argentine data. For reference, an uninformative (random) classifier has AUC = 0.5 and a perfect classifier has AUC = 1.0. The total number of correctly called genotypes (non-error genotypes) and incorrectly called (error) genotypes are shown in the rightmost columns.
