# Supplementary material for: GBStools: A Statistical Method for Estimating Allelic Dropout in Reduced Representation Sequencing Data
Source: PLoS Genet. 2016 Feb 1;12(2):e1005631. doi: 10.1371/journal.pgen.1005631 (PMC4734769; doi:10.1371/journal.pgen.1005631)
Supplement: S4 Table — Read pairs are given per million (M), and coverage is given in fold-coverage, the final column indicates whether the samples were included (Y) or excluded (N) from the downstream analysis. The target region is defined to be the union of simulated digest fragments between 200–600 bp that had ≥ 3X mean coverage per sample and where ≥ 10% of mate pairs were mapped to restriction sites at the end of the fragment. (PDF) [file pgen.1005631.s013.pdf]

| | | | | Mapped to Restriction Sites: | | | | |
|---|---|---|---|---|---|---|---|---|
| Sample | Read pairs (M) | Mapped pairs (%) | On target pairs (%) | 0 of 2 in pair (%) | 1 of 2 In pair (%) | 2 of 2 In pair (%) | Coverage in target | Kept |
| Mj0722 | 7.0 | 96.6 | 47.2 | 15.4 | 32.7 | 51.9 | 4.0 | Y |
| Me0395 | 23.8 | 97.1 | 47.5 | 16.5 | 34.3 | 49.2 | 12.2 | Y |
| Mb0125 | 18.1 | 96.4 | 42.2 | 21.0 | 37.2 | 41.9 | 8.3 | Y |
| Mi0652 | 7.4 | 96.0 | 41.9 | 24.1 | 37.4 | 38.5 | 4.0 | Y |
| Mg0523 | 13.6 | 96.7 | 37.1 | 22.8 | 39.7 | 37.5 | 5.6 | Y |
| Ma0001 | 46.8 | 96.2 | 46.1 | 20.5 | 42.6 | 36.9 | 22.8 | Y |
| X1 | 21.3 | 97.0 | 36.6 | 24.3 | 40.3 | 35.4 | 8.5 | Y |
| Mg0520 | 19.1 | 95.4 | 44.4 | 23.6 | 42.6 | 33.8 | 9.1 | Y |
| Mj0721 | 23.5 | 94.5 | 49.5 | 22.5 | 44.1 | 33.5 | 12.3 | Y |
| X4 | 28.0 | 96.7 | 35.4 | 25.7 | 41.4 | 33.0 | 10.6 | Y |
| Mn0843 | 20.5 | 93.7 | 53.7 | 24.2 | 43.5 | 32.3 | 11.8 | Y |
| Mc0250 | 10.7 | 95.7 | 47.9 | 22.9 | 45.7 | 31.4 | 5.7 | Y |
| Md0336 | 7.8 | 95.6 | 49.6 | 23.7 | 45.8 | 30.6 | 4.5 | Y |
| Mo0877 | 33.7 | 96.7 | 36.8 | 25.4 | 44.7 | 29.9 | 13.3 | Y |
| Mn0842 | 19.6 | 93.5 | 47.3 | 26.9 | 44.3 | 28.8 | 9.9 | Y |
| Mc0254 | 16.5 | 96.4 | 46.4 | 25.1 | 46.4 | 28.5 | 8.2 | Y |
| Ml0793 | 10.4 | 97.1 | 37.3 | 27.6 | 44.8 | 27.7 | 4.5 | Y |
| Mb0121 | 8.1 | 96.1 | 41.7 | 26.8 | 46.4 | 26.8 | 4.3 | Y |
| X5 | 21.4 | 96.8 | 35.6 | 29.3 | 44.3 | 26.4 | 8.2 | Y |
| Mb0124 | 12.0 | 96.4 | 45.7 | 27.1 | 46.7 | 26.3 | 6.1 | Y |
| Ml0780 | 25.0 | 94.1 | 43.4 | 29.2 | 45.4 | 25.5 | 11.5 | Y |
| Mb0122 | 8.1 | 96.3 | 42.4 | 29.0 | 46.5 | 24.5 | 4.1 | Y |
| Mh0607 | 19.4 | 91.7 | 42.8 | 32.1 | 43.5 | 24.4 | 9.0 | Y |
| Ma0002 | 20.1 | 93.9 | 44.4 | 30.6 | 45.1 | 24.4 | 9.8 | Y |
| Mc0246 | 26.6 | 93.5 | 46.3 | 31.7 | 44.1 | 24.2 | 13.0 | Y |
| Ml0781 | 21.0 | 93.4 | 42.0 | 31.4 | 44.7 | 23.9 | 9.4 | Y |
| X3 | 20.3 | 96.6 | 32.8 | 32.7 | 43.4 | 23.9 | 7.1 | Y |
| Mo0878 | 31.1 | 93.1 | 42.9 | 32.8 | 43.9 | 23.3 | 14.1 | Y |
| Ml0796 | 17.7 | 96.8 | 34.9 | 32.4 | 44.7 | 22.9 | 6.8 | Y |
| Mi0640 | 27.9 | 88.9 | 42.9 | 35.3 | 41.8 | 22.9 | 12.7 | Y |
| Md0337 | 10.4 | 94.5 | 42.4 | 31.4 | 45.7 | 22.9 | 5.1 | Y |
| Ma0004 | 9.7 | 90.1 | 33.4 | 37.6 | 39.9 | 22.5 | 4.7 | Y |
| Mm0811 | 14.9 | 97.0 | 34.7 | 33.5 | 44.3 | 22.1 | 5.8 | Y |
| Mo0879 | 21.5 | 97.0 | 28.3 | 34.3 | 43.6 | 22.1 | 6.6 | Y |
| Md0334 | 23.4 | 87.4 | 45.5 | 36.5 | 41.8 | 21.7 | 11.4 | Y |
| Md0340 | 6.1 | 94.6 | 42.8 | 32.4 | 46.1 | 21.6 | 3.4 | Y |
| Mk0757 | 20.2 | 97.0 | 33.9 | 34.3 | 44.2 | 21.5 | 7.5 | Y |
| Me0392 | 7.6 | 94.8 | 39.8 | 33.3 | 45.8 | 20.9 | 3.7 | Y |
| Mf0447 | 19.1 | 92.1 | 43.1 | 35.1 | 44.3 | 20.6 | 8.9 | Y |
| Me0396 | 17.7 | 96.7 | 27.2 | 35.8 | 43.9 | 20.3 | 5.4 | Y |
| Me0391 | 17.6 | 94.4 | 40.0 | 35.9 | 44.5 | 19.5 | 7.7 | Y |
| Mi0646 | 8.8 | 94.4 | 29.9 | 43.1 | 37.8 | 19.1 | 3.2 | Y |
| Mg0521 | 22.0 | 90.6 | 42.7 | 37.8 | 43.3 | 18.9 | 10.2 | Y |
| Mn0848 | 20.6 | 97.0 | 26.0 | 37.1 | 44.0 | 18.8 | 5.9 | Y |
| Mi0641 | 38.4 | 83.7 | 38.8 | 42.8 | 38.7 | 18.6 | 15.9 | Y |
| Mf0453 | 17.9 | 93.7 | 30.2 | 43.6 | 38.5 | 17.9 | 5.9 | Y |
| Mf0448 | 19.5 | 91.7 | 40.3 | 38.4 | 43.8 | 17.8 | 8.6 | Y |
| Mm0820 | 14.5 | 94.8 | 30.8 | 40.0 | 42.4 | 17.5 | 5.0 | Y |
| Mh0620 | 9.6 | 94.6 | 30.2 | 41.4 | 41.8 | 16.7 | 3.5 | Y |
| Mo0882 | 5.7 | 94.9 | 29.9 | 42.1 | 41.4 | 16.4 | 2.5 | Y |
| Mb0123 | 7.1 | 96.2 | 34.5 | 37.5 | 46.2 | 16.3 | 3.3 | Y |
| Mc0249 | 9.6 | 94.4 | 36.2 | 38.1 | 45.6 | 16.2 | 4.3 | Y |
| Me0390 | 22.5 | 92.0 | 33.6 | 41.7 | 42.4 | 15.9 | 8.3 | Y |
| Mc0248 | 13.7 | 94.6 | 37.9 | 38.5 | 45.8 | 15.7 | 6.0 | Y |
| Me0393 | 9.4 | 93.3 | 35.4 | 39.8 | 44.8 | 15.3 | 4.1 | Y |
| Mk0754 | 9.0 | 95.9 | 25.8 | 45.7 | 39.9 | 14.4 | 3.1 | Y |
| Mm0812 | 17.2 | 95.7 | 27.3 | 50.4 | 35.9 | 13.7 | 5.5 | Y |
| Mf0450 | 9.1 | 88.9 | 27.0 | 52.5 | 34.5 | 13.0 | 3.2 | Y |
| Mf0449 | 7.8 | 91.9 | 26.7 | 51.6 | 35.5 | 12.9 | 2.8 | Y |
| Mj0732 | 17.4 | 96.8 | 25.7 | 45.5 | 41.9 | 12.6 | 4.9 | Y |
| Md0335 | 18.9 | 87.9 | 30.5 | 48.4 | 39.4 | 12.2 | 6.6 | Y |
| Mj0720 | 28.5 | 86.5 | 31.8 | 48.3 | 40.1 | 11.6 | 10.1 | Y |
| Mc0247 | 21.0 | 90.0 | 29.7 | 49.6 | 39.3 | 11.1 | 7.3 | Y |
| Mh0606 | 1.3 | 92.4 | 36.9 | 42.4 | 43.6 | 14.0 | 1.5 | N |
| Ml0782 | 8.7 | 94.0 | 25.2 | 53.9 | 32.4 | 13.8 | 2.9 | N |
| Mi0656 | 16.4 | 91.6 | 26.0 | 53.6 | 35.0 | 11.4 | 4.9 | N |
| Mn0846 | 17.7 | 89.7 | 24.6 | 57.5 | 32.5 | 10.0 | 5.1 | N |
| Mh0612 | 7.6 | 90.7 | 19.7 | 68.3 | 22.9 | 8.8 | 2.3 | N |
| Mi0642 | 7.9 | 89.0 | 18.0 | 73.6 | 17.9 | 8.6 | 2.2 | N |
| X6 | 18.3 | 95.2 | 20.1 | 55.0 | 36.9 | 8.2 | 4.1 | N |
| Ma0012 | 15.8 | 90.9 | 22.3 | 60.2 | 31.7 | 8.1 | 4.3 | N |
| Mg0530 | 10.6 | 87.8 | 19.6 | 70.4 | 21.7 | 8.0 | 2.9 | N |
| Mh0609 | 6.2 | 87.8 | 19.8 | 68.4 | 23.7 | 7.9 | 2.2 | N |
| X2 | 19.5 | 97.0 | 20.8 | 55.5 | 36.7 | 7.8 | 4.5 | N |
| Me0394 | 9.0 | 85.4 | 18.8 | 71.6 | 21.6 | 6.8 | 2.9 | N |
| Mf0452 | 12.7 | 86.7 | 18.5 | 72.2 | 21.3 | 6.5 | 3.2 | N |
| Mg0522 | 8.5 | 87.1 | 17.3 | 73.9 | 19.8 | 6.2 | 2.5 | N |
| Ma0010 | 8.6 | 87.1 | 20.0 | 68.8 | 25.2 | 6.0 | 2.8 | N |
| Mh0608 | 7.7 | 88.9 | 15.9 | 76.2 | 18.5 | 5.3 | 2.2 | N |
| Mk0756 | 13.0 | 94.5 | 15.5 | 77.9 | 17.3 | 4.9 | 3.0 | N |
| Mn0845 | 8.5 | 88.0 | 16.1 | 77.0 | 18.1 | 4.8 | 2.3 | N |
| Mk0764 | 9.2 | 89.5 | 15.9 | 76.6 | 19.5 | 4.0 | 2.5 | N |
| Mg0529 | 12.7 | 89.7 | 13.0 | 82.0 | 14.4 | 3.6 | 2.6 | N |
| Mi0649 | 11.1 | 85.5 | 14.4 | 78.0 | 18.4 | 3.6 | 2.7 | N |
| Mh0615 | 14.9 | 95.4 | 12.3 | 76.5 | 20.1 | 3.4 | 2.5 | N |
| Mj0724 | 6.8 | 91.3 | 12.8 | 85.5 | 11.6 | 2.9 | 2.1 | N |
| Ma0006 | 10.9 | 88.9 | 12.3 | 83.7 | 14.3 | 2.1 | 2.2 | N |
| Mj0733 | 7.9 | 90.2 | 11.9 | 84.4 | 13.8 | 1.7 | 1.8 | N |
| Mk0753 | 6.4 | 92.1 | 9.5 | 91.2 | 7.3 | 1.5 | 1.7 | N |
| Mean (kept) | 17.5 | 94.2 | 38.0 | 33.8 | 42.5 | 23.8 | 7.5 | — |
| Mean (all) | 15.5 | 93.0 | 32.3 | 44.5 | 36.8 | 18.7 | 6.1 | — |

**S4 Table. Read mapping results for Argentine individuals.** Read pairs are given per million (M), and coverage is given in fold-coverage, the final column indicates whether the samples were included (Y) or excluded (N) from the downstream analysis. The target region is defined to be the union of simulated digest fragments between 200-600 bp that had ≥ 3X mean coverage per sample and where ≥ 10% of mate pairs were mapped to restriction sites at the end of the fragment.
