# Supplementary material for: GBStools: A Statistical Method for Estimating Allelic Dropout in Reduced Representation Sequencing Data
Source: PLoS Genet. 2016 Feb 1;12(2):e1005631. doi: 10.1371/journal.pgen.1005631 (PMC4734769; doi:10.1371/journal.pgen.1005631)
Supplement: S5 Table — Oligo sequence and barcode index for 65 sequencing adaptors used for library preparation. Adapters were made by annealing each of the index adapters #1–65 to the common adapter (methods). (PDF) [file pgen.1005631.s014.pdf]

Index	Sequence	i7 index	i7 sample sheet entry
common adapter	5'-AATGATACGGCGACCACCGAGATCTACACTCTTTCCCTACACGACGCTCTTCCGATCT	-	-
1	5'-phosphate-GATCGGAAGAGCACACGTCTGAACTCCAGTCACCGTGATATCTCGTATGCCGTCTTCTGCTTG	CGTGAT	ATCACG
2	5'-phosphate-GATCGGAAGAGCACACGTCTGAACTCCAGTCACACATCGATCTCGTATGCCGTCTTCTGCTTG	ACATCG	CGATGT
3	5'-phosphate-GATCGGAAGAGCACACGTCTGAACTCCAGTCACGCCTAAATCTCGTATGCCGTCTTCTGCTTG	GCCTAA	TTAGGC
4	5'-phosphate-GATCGGAAGAGCACACGTCTGAACTCCAGTCACTGGTCAATCTCGTATGCCGTCTTCTGCTTG	TGGTCA	TGACCA
5	5'-phosphate-GATCGGAAGAGCACACGTCTGAACTCCAGTCACCACTGTATCTCGTATGCCGTCTTCTGCTTG	CACTGT	ACAGTG
6	5'-phosphate-GATCGGAAGAGCACACGTCTGAACTCCAGTCACATTGGCATCTCGTATGCCGTCTTCTGCTTG	ATTGGC	GCCAAT
7	5'-phosphate-GATCGGAAGAGCACACGTCTGAACTCCAGTCACGATCTGATCTCGTATGCCGTCTTCTGCTTG	GATCTG	CAGATC
8	5'-phosphate-GATCGGAAGAGCACACGTCTGAACTCCAGTCACTCAAGTATCTCGTATGCCGTCTTCTGCTTG	TCAAGT	ACTTGA
9	5'-phosphate-GATCGGAAGAGCACACGTCTGAACTCCAGTCACCTGATCATCTCGTATGCCGTCTTCTGCTTG	CTGATC	GATCAG
10	5'-phosphate-GATCGGAAGAGCACACGTCTGAACTCCAGTCACAAGCTAATCTCGTATGCCGTCTTCTGCTTG	AAGCTA	TAGCTT
11	5'-phosphate-GATCGGAAGAGCACACGTCTGAACTCCAGTCACGTAGCCATCTCGTATGCCGTCTTCTGCTTG	GTAGCC	GGCTAC
12	5'-phosphate-GATCGGAAGAGCACACGTCTGAACTCCAGTCACTACAAGATCTCGTATGCCGTCTTCTGCTTG	TACAAG	CTTGTA
13	5'-phosphate-GATCGGAAGAGCACACGTCTGAACTCCAGTCACTCGATGATCTCGTATGCCGTCTTCTGCTTG	TCGATG	CATCGA
14	5'-phosphate-GATCGGAAGAGCACACGTCTGAACTCCAGTCACGGTCTAATCTCGTATGCCGTCTTCTGCTTG	GGTCTA	TAGACC
15	5'-phosphate-GATCGGAAGAGCACACGTCTGAACTCCAGTCACTGGATCATCTCGTATGCCGTCTTCTGCTTG	TGGATC	GATCCA
16	5'-phosphate-GATCGGAAGAGCACACGTCTGAACTCCAGTCACTGCTAGATCTCGTATGCCGTCTTCTGCTTG	TGCTAG	CTAGCA
17	5'-phosphate-GATCGGAAGAGCACACGTCTGAACTCCAGTCACTAAGCCATCTCGTATGCCGTCTTCTGCTTG	TAAGCC	GGCTTA
18	5'-phosphate-GATCGGAAGAGCACACGTCTGAACTCCAGTCACGTCAGTATCTCGTATGCCGTCTTCTGCTTG	GTCAGT	ACTGAC
19	5'-phosphate-GATCGGAAGAGCACACGTCTGAACTCCAGTCACCCAGATATCTCGTATGCCGTCTTCTGCTTG	CCAGAT	ATCTGG
20	5'-phosphate-GATCGGAAGAGCACACGTCTGAACTCCAGTCACGACTGTATCTCGTATGCCGTCTTCTGCTTG	GACTGT	ACAGTC
21	5'-phosphate-GATCGGAAGAGCACACGTCTGAACTCCAGTCACCACAGTATCTCGTATGCCGTCTTCTGCTTG	CACAGT	ACTGTG
22	5'-phosphate-GATCGGAAGAGCACACGTCTGAACTCCAGTCACGCAGTTATCTCGTATGCCGTCTTCTGCTTG	GCAGTT	AACTGC
23	5'-phosphate-GATCGGAAGAGCACACGTCTGAACTCCAGTCACCTCAGAATCTCGTATGCCGTCTTCTGCTTG	CTCAGA	TCTGAG
24	5'-phosphate-GATCGGAAGAGCACACGTCTGAACTCCAGTCACGTCTGAATCTCGTATGCCGTCTTCTGCTTG	GTCTGA	TCAGAC
25	5'-phosphate-GATCGGAAGAGCACACGTCTGAACTCCAGTCACGGTCATATCTCGTATGCCGTCTTCTGCTTG	GGTCAT	ATGACC
26	5'-phosphate-GATCGGAAGAGCACACGTCTGAACTCCAGTCACACGTACATCTCGTATGCCGTCTTCTGCTTG	ACGTAC	GTACGT
27	5'-phosphate-GATCGGAAGAGCACACGTCTGAACTCCAGTCACAGCATCATCTCGTATGCCGTCTTCTGCTTG	AGCATC	GATGCT
28	5'-phosphate-GATCGGAAGAGCACACGTCTGAACTCCAGTCACGCACATATCTCGTATGCCGTCTTCTGCTTG	GCACAT	ATGTGC
29	5'-phosphate-GATCGGAAGAGCACACGTCTGAACTCCAGTCACACGTTGATCTCGTATGCCGTCTTCTGCTTG	ACGTTG	CAACGT
30	5'-phosphate-GATCGGAAGAGCACACGTCTGAACTCCAGTCACCCTGAAATCTCGTATGCCGTCTTCTGCTTG	CCTGAA	TTCAGG
31	5'-phosphate-GATCGGAAGAGCACACGTCTGAACTCCAGTCACTGCAACATCTCGTATGCCGTCTTCTGCTTG	TGCAAC	GTTGCA
32	5'-phosphate-GATCGGAAGAGCACACGTCTGAACTCCAGTCACAGCTTGATCTCGTATGCCGTCTTCTGCTTG	AGCTTG	CAAGCT
33	5'-phosphate-GATCGGAAGAGCACACGTCTGAACTCCAGTCACGTGTCAATCTCGTATGCCGTCTTCTGCTTG	GTGTCA	TGACAC
34	5'-phosphate-GATCGGAAGAGCACACGTCTGAACTCCAGTCACTCGAACATCTCGTATGCCGTCTTCTGCTTG	TCGAAC	GTTCGA
35	5'-phosphate-GATCGGAAGAGCACACGTCTGAACTCCAGTCACCGAGTTATCTCGTATGCCGTCTTCTGCTTG	CGAGTT	AACTCG
36	5'-phosphate-GATCGGAAGAGCACACGTCTGAACTCCAGTCACACCATGATCTCGTATGCCGTCTTCTGCTTG	ACCATG	CATGGT
37	5'-phosphate-GATCGGAAGAGCACACGTCTGAACTCCAGTCACCACTGAATCTCGTATGCCGTCTTCTGCTTG	CACTGA	TCAGTG
38	5'-phosphate-GATCGGAAGAGCACACGTCTGAACTCCAGTCACCGTCAAATCTCGTATGCCGTCTTCTGCTTG	CGTCAA	TTGACG
39	5'-phosphate-GATCGGAAGAGCACACGTCTGAACTCCAGTCACAGCTACATCTCGTATGCCGTCTTCTGCTTG	AGCTAC	GTAGCT
40	5'-phosphate-GATCGGAAGAGCACACGTCTGAACTCCAGTCACGTGACTATCTCGTATGCCGTCTTCTGCTTG	GTGACT	AGTCAC
41	5'-phosphate-GATCGGAAGAGCACACGTCTGAACTCCAGTCACCCAGTAATCTCGTATGCCGTCTTCTGCTTG	CCAGTA	TACTGG
42	5'-phosphate-GATCGGAAGAGCACACGTCTGAACTCCAGTCACGCTCAAATCTCGTATGCCGTCTTCTGCTTG	GCTCAA	TTGAGC
43	5'-phosphate-GATCGGAAGAGCACACGTCTGAACTCCAGTCACTCGTAGATCTCGTATGCCGTCTTCTGCTTG	TCGTAG	CTACGA
44	5'-phosphate-GATCGGAAGAGCACACGTCTGAACTCCAGTCACCAGACTATCTCGTATGCCGTCTTCTGCTTG	CAGACT	AGTCTG
45	5'-phosphate-GATCGGAAGAGCACACGTCTGAACTCCAGTCACAGGTTCATCTCGTATGCCGTCTTCTGCTTG	AGGTTC	GAACCT
46	5'-phosphate-GATCGGAAGAGCACACGTCTGAACTCCAGTCACCGACTAATCTCGTATGCCGTCTTCTGCTTG	CGACTA	TAGTCG
47	5'-phosphate-GATCGGAAGAGCACACGTCTGAACTCCAGTCACGCACTAATCTCGTATGCCGTCTTCTGCTTG	GCACTA	TAGTGC
48	5'-phosphate-GATCGGAAGAGCACACGTCTGAACTCCAGTCACACCTAGATCTCGTATGCCGTCTTCTGCTTG	ACCTAG	CTAGGT
49	5'-phosphate-GATCGGAAGAGCACACGTCTGAACTCCAGTCACGAGTCTATCTCGTATGCCGTCTTCTGCTTG	GAGTCT	AGACTC
50	5'-phosphate-GATCGGAAGAGCACACGTCTGAACTCCAGTCACGTCACAATCTCGTATGCCGTCTTCTGCTTG	GTCACA	TGTGAC
51	5'-phosphate-GATCGGAAGAGCACACGTCTGAACTCCAGTCACTGGTACATCTCGTATGCCGTCTTCTGCTTG	TGGTAC	GTACCA
52	5'-phosphate-GATCGGAAGAGCACACGTCTGAACTCCAGTCACGCTGATATCTCGTATGCCGTCTTCTGCTTG	GCTGAT	ATCAGC
53	5'-phosphate-GATCGGAAGAGCACACGTCTGAACTCCAGTCACGGACTTATCTCGTATGCCGTCTTCTGCTTG	GGACTT	AAGTCC
54	5'-phosphate-GATCGGAAGAGCACACGTCTGAACTCCAGTCACTGCATGATCTCGTATGCCGTCTTCTGCTTG	TGCATG	CATGCA
55	5'-phosphate-GATCGGAAGAGCACACGTCTGAACTCCAGTCACCAGTCAATCTCGTATGCCGTCTTCTGCTTG	CAGTCA	TGACTG
56	5'-phosphate-GATCGGAAGAGCACACGTCTGAACTCCAGTCACGACACTATCTCGTATGCCGTCTTCTGCTTG	GACACT	AGTGTC
57	5'-phosphate-GATCGGAAGAGCACACGTCTGAACTCCAGTCACCTGTGAATCTCGTATGCCGTCTTCTGCTTG	CTGTGA	TCACAG
58	5'-phosphate-GATCGGAAGAGCACACGTCTGAACTCCAGTCACCGACATATCTCGTATGCCGTCTTCTGCTTG	CGACAT	ATGTCG
59	5'-phosphate-GATCGGAAGAGCACACGTCTGAACTCCAGTCACGACTCAATCTCGTATGCCGTCTTCTGCTTG	GACTCA	TGAGTC
60	5'-phosphate-GATCGGAAGAGCACACGTCTGAACTCCAGTCACCGTGTAATCTCGTATGCCGTCTTCTGCTTG	CGTGTA	TACACG
61	5'-phosphate-GATCGGAAGAGCACACGTCTGAACTCCAGTCACACGATCATCTCGTATGCCGTCTTCTGCTTG	ACGATC	GATCGT
62	5'-phosphate-GATCGGAAGAGCACACGTCTGAACTCCAGTCACTCCAAGATCTCGTATGCCGTCTTCTGCTTG	TCCAAG	CTTGGA
63	5'-phosphate-GATCGGAAGAGCACACGTCTGAACTCCAGTCACCTGACAATCTCGTATGCCGTCTTCTGCTTG	CTGACA	TGTCAG
64	5'-phosphate-GATCGGAAGAGCACACGTCTGAACTCCAGTCACGCTGTAATCTCGTATGCCGTCTTCTGCTTG	GCTGTA	TACAGC
65	5'-phosphate-GATCGGAAGAGCACACGTCTGAACTCCAGTCACCTGAGTATCTCGTATGCCGTCTTCTGCTTG	CTGAGT	ACTCAG

S5 Table. Sequencing adapters. Oligo sequence and barcode index for 65 sequencing adaptors used for library preparation. Adapters were made by annealing each of the index adapters #1-65 to the common adapter (methods).
